# Serum Paraoxonase-1-Related Variables and Lipoprotein Profile in Patients with Lung or Head and Neck Cancer: Effect of Radiotherapy

**DOI:** 10.3390/antiox8070213

**Published:** 2019-07-10

**Authors:** Elisabet Rodríguez-Tomàs, Mauricio Murcia, Meritxell Arenas, Mònica Arguís, Miriam Gil, Núria Amigó, Xavier Correig, Laura Torres, Sebastià Sabater, Gerard Baiges-Gayà, Noemí Cabré, Fedra Luciano-Mateo, Anna Hernández-Aguilera, Isabel Fort-Gallifa, Jordi Camps, Jorge Joven

**Affiliations:** 1 Universitat Rovira i Virgili, Departament de Medicina i Cirurgia, Unitat de Recerca Biomèdica, 43201 Reus, Spain; 2 Unitat de Recerca Biomèdica, Hospital Universitari Sant Joan de Reus, Institut d’Investigació Sanitària Pere Virgili, 43201 Reus, Spain; 3 Department of Radiation Oncology, Hospital Universitari Sant Joan de Reus, Institut d’Investigació Sanitària Pere Virgili, Universitat Rovira i Virgili, 43201 Reus, Spain; 4 Biosfer Teslab, 43201 Reus, Spain; 5 Metabolomics Platform, CIBERDEM, Institut d’Investigació Sanitària Pere Virgili, Universitat Rovira i Virgili, 43201 Reus, Spain

**Keywords:** antioxidants, head and neck cancer, lipoproteins, lung cancer, paraoxonase-1, radiotherapy

## Abstract

We investigated alterations in the levels of the antioxidant paraoxonase-1 (PON1) and the lipoprotein profile (analyzed by nuclear magnetic resonance) in patients with lung cancer (LC) or head and neck cancer (HNC), and the effects produced thereon by radiotherapy (RT). We included 33 patients with LC and 28 patients with HNC. Before irradiation, and one month after completion of RT, blood samples were obtained. The control group was composed of 50 healthy subjects. Patients had significantly lower serum PON1 activity and concentration before RT than the control group. PON1-related variables were good predictors of the presence of LC or HNC, with analytical sensitivities and specificities greater than 80%. Patients showed a significant increase in the number of particles of all subclasses of very-low-density lipoproteins (large, medium and small). However, these changes were not maintained when adjusted for age, sex, and other clinical and demographic variables. Irradiation was associated with a significant increase in PON1 concentration and, only in patients with HNC, with an increase in high-density lipoprotein-cholesterol concentration. Our results suggest that determinations of the levels of PON1-related variables may constitute good biomarkers for the evaluation of these diseases. Studies with a larger number of patients are needed to fully confirm this hypothesis.

## 1. Introduction

Lung cancer (LC) and head and neck cancer (HNC) are among the most common malignant diseases in the world, and are the leading causes of cancer-related death in both men and women [1,2]. Genetic susceptibility, lifestyle factors, and environmental agents are important contributors to the etiology of LC or HNC. Both types of cancer share tobacco smoking as a carcinogen that strongly contributes to their etiology. One of the deleterious effects of smoking is an increase in oxidative stress. This derangement and consequent lipid peroxidation are involved in the oncogenesis process [3]. Moreover, it has been observed that oxidized low-density lipoproteins (LDL) were are associated with oxidative stress-related cancers [4]. Recent studies suggest that paraoxonase-1 (PON1) plays a significant role in the pathophysiology of malignant diseases. PON1 is an antioxidant enzyme that degrades oxidized lipids in lipoproteins and cells. This enzyme is synthesized mainly by the liver, and is found in the membranes of many cell types, particularly in the epithelia, as well as in the circulation bound to high-density lipoproteins (HDL) [5,6]. The original function attributed to PON1 is thought to be lipolactonase [7]; it is this catalytic capacity that enables the enzyme to degrade lipid peroxides. Several studies have reported that a His115-His134 dyad is necessary for PON1 lactonase activity, as well as for the degradation of oxidized lipids. The antioxidant function of this enzyme seems to involve the lactonization of oxidized lipids containing hydroxyl groups at the 5′-position, yielding lysophosphatidylcholine and δ-valerolactone products which are hydrolyzed again by the enzyme [8]. In addition, PON1 has esterase activity and degrades organophosphate xenobiotics such as paraoxon (C_10_H_14_N_1_O_6_P_1_), phenyl acetate (C_8_H_8_O_2_) and nerve agents [6]. Several studies have reported a decrease in serum PON1 activity in LC or HNC patients [3,9], and people exposed to organophosphate insecticides have a higher incidence of LC than the non-exposed population [10]. However, PON1 is not the only component of the HDL particles to have been associated with cancer. Indeed, low HDL-cholesterol concentrations have been reported to be a risk factor for LC [11]. Recent nuclear magnetic resonance (NMR) methods have allowed researchers to perform a detailed characterization of lipoprotein particles and obtain more information than with conventional biochemical methods [12].

The current preferred treatment of LC and HNC cancers is radiotherapy (RT) combined with chemotherapy. RT is an effective oncological treatment, but it can produce different physiological responses due to disease heterogeneity and sensitivity to treatment. Different studies have shown that metabolism plays a critical role during oncogenesis development. However, there is still a lack of information about the metabolic response to RT. If the effects of RT on tumor cells and changes on patient metabolism were better known, specialists would be able to improve the planning and follow-up of these patients. Nowadays, there is a paucity of studies on the effects of RT on PON1-related variables and lipoprotein characteristics in patients with LC or HNC.

The aims of our study were to investigate the alterations in serum PON1 activity and concentration and the lipoprotein profile (analyzed by NMR) in LC and HNC in order to relate these changes with the clinical and pathological characteristics of these patients and their response to treatment, and to determine the effects produced by RT on these parameters.

## 2. Materials and Methods

We included 33 patients diagnosed as having LC (78% male; age: 65–79 years old) and 28 patients diagnosed as having HNC (89% male; age: 56–73 years old), which attended the Department of Radiation Oncology of our Hospital. The Karnofsky Index was higher than 70 in all patients, who were classified using the Eastern Cooperative Oncology Group scale (0 or 1) [13]. Patients that had previously received RT at the same cancer site or were pregnant or breastfeeding were excluded. LC patients were diagnosed as having non-small cell lung cancer needing radical RT treatment. The radiation schedule was normofractionated RT (total dose 60–66 Gy at 2 Gy/day, 5 days/week) for LC, and normofractionated loco-regional RT (total dose 60–70 Gy according to the stage, at 2 Gy/day, 5 days/week), for HNC, using the Volumetric Modulated Arc Therapy method (Varian RapidArc®, Varian Medical Systems, Palo Alto, CA, USA). Criteria from Radiation Therapy Oncology Group and the European Organization for Research and Treatment of Cancer were used to weekly assess acute toxicity during RT [14]. Before and one month after irradiation, sera and EDTA-plasma samples were obtained and immediately stored at −80 °C until used for biochemical analyses. Fourteen LC patients were also treated with cisplatin (50 mg/m^2^) and etoposide (50 mg/m^2^) IV every 3 weeks, and 11 HNC patients received cisplatin (100 mg/m^2^) IV every 3 weeks concomitant to radiation therapy. A detailed description of the clinical characteristics of the LC or HNC patients and their treatments is shown in Table 1.

Patients were followed for up to one year, and response-to-treatment was assessed by computed tomography scan or magnetic resonance imaging. In some patients, assessments were complemented with positron emission tomography. Complete response (CR) was defined as the disappearance of all lesions and lymph nodes <10 mm. Partial response (PR) was defined as a ≥30% decrease in the sum of target disease compared with baseline scan. Progression of disease (PD) comprised an increase in the sum of ≥20% and >0 5 mm compared with the smallest sum obtained during follow-up. Disease-free survival (DFS) was defined as the time from the diagnosis to recurrence and/or metastasis, whichever was earliest. Loco-regional progression of the disease (LPD) was defined as a time elapsed between diagnosis and loco-regional recurrence or death dates. Distant progression of the disease (DD) was defined as a time elapsed between diagnosis and metastasis or death dates [15].

The control group was composed of 50 healthy subjects (50% male; age: 35–47 years old) participating in a population-based study conducted in our geographical area. The study was approved by the Ethics Committee (Institutional Review Board) of the Hospital Universitari de Sant Joan (project code: 14/2017). Written informed consents were obtained from all patients according to the declaration of Helsinki. Exclusion criteria included evidence of infectious disease, renal insufficiency, hepatic damage, neoplasia, or mental illness [16].

Serum PON1 esterase activity was measured by analyzing the hydrolysis of phenyl acetate (arylesterase activity, ARE), as previously described [17]. An in-house, enzyme-linked, immunosorbent assay (ELISA) was used to determine serum PON1 concentration with a rabbit polyclonal antibody generated against the synthetic peptide CRNHQSSYQTRLNALREVQ, which is a specific sequence for mature PON1 [18]. The ARE specific activity is an estimate of the enzymatic activity per molecule of PON1, and was calculated as the ratio between the respective activities and concentrations. Total blood hemoglobin and platelets were measured in a Sysmex XN analyzer (Roche Diagnostics, Risch-Rotkreuz, Switzerland).

The Liposcale Test was used to determine lipid content, particle number and size of lipoproteins in plasma samples by NMR [12]. The methyl signal of 2D ^1^H-NMR spectra was deconvoluted with lorentzian functions corresponding to 9 subclasses, i.e., large, medium and small, of main lipoprotein classes (VLDL, LDL and HDL). Lipid content was obtained from the area of each function whereas diffusion coefficient of each function was associated with lipoprotein particle size. To determine lipid volumes, common conversion factors were used [19]. Finally, data from lipid content and lipoprotein particle size were combined to determine particle number of each lipoprotein subclass.

A Kolmogorov-Smirnov test was used to assess the distribution characteristics of variables. Mann-Whitney *U*-test (non-parametric) was used to assess differences between any two groups of variables. The Spearman correlation coefficient was used to evaluate the degree of association between quantitative variables. The χ-square test was employed to evaluate differences in qualitative variables. Binary logistic regression analysis was employed to investigate the independent association between clinical and demographic characteristics, PON1-related variables and the presence or absence of disease. The diagnostic accuracy of measured biochemical variables was assessed by receiver operating characteristics (ROC) curve which employs plots of sensitivity/specificity based on decision thresholds. Sensitivity (or true positive rate) represents those samples correctly identified as disease-associated. Specificity (or true negative rate) represents those subjects correctly identified as not being affected by a specific disease. The false positive rate was calculated as 1-specificity. The area under the curve (AUC) and 95% confidence interval (CI) values were also determined. The AUC represents the capacity of the compound to correctly distinguish between patients with or without the investigated alteration. The values of AUC can vary between 1 (perfect test) and 0.5 (worthless test) [20]. For some biochemical measurements, we performed a multivariate analysis of pattern recognition, including the unsupervised principal component analysis (PCA) and the supervised partial least squares discriminant analysis (PLSDA). Variable importance in the projection (VIP) score was used to test the relative significance of obtained results [21]. All statistical analyses and related graphics were obtained with GraphPad Prism software 6.01 (GraphPad Software, San Diego, CA, USA), SPSS Software (IBM SPSS Statistics for Windows, Version 25.00 Armonk, NY.) and MetaboAnalyst 4.0 (www.metaboanalyst.ca). Statistically significant differences were considered when the *p* value was ≤0.05.

## 3. Results

### 3.1. Changes in PON1-Related Variables and Lipoprotein Profile in Patients with LC or HNC

**Figure 1:**
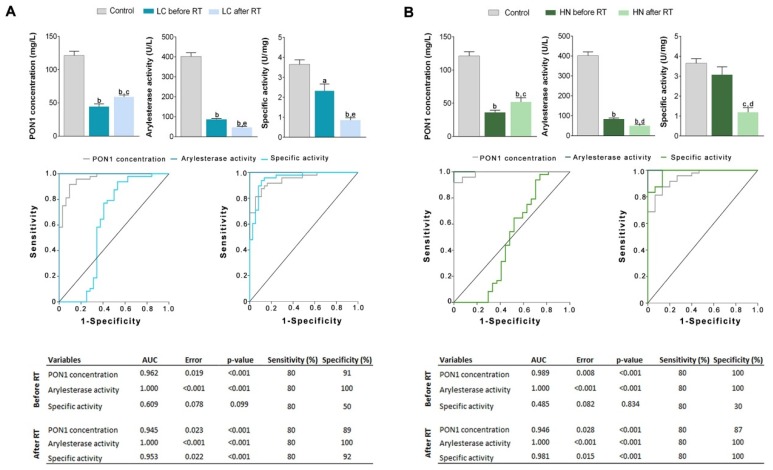
Serum paraoxonase-1 (PON1) concentration, arylesterase (ARE) activity, PON1 specific activity, and receiver operating characteristics (ROC) curves in patients with (**A**) lung cancer (LC) or (**B**) head and neck cancer (HNC) before and after radiotherapy (RT). Results are shown as means and standard errors. ^a^ *p* < 0.01, ^b^ *p* < 0.001, with respect to the control group; ^c^ *p* < 0.05, ^d^ *p* < 0.01, ^e^ *p* < 0.001, with respect to pre-radiotherapy, by the Mann-Whitney *U*-test.

Both types of patients had significantly lower serum ARE activity and PON1 concentration before RT than the control group. Irradiation was associated with a significant increase in PON1 concentration and a further decrease in the enzymatic activity. Consequently, specific activity after RT was lower than before. ROC plots showed that PON1 concentration and ARE activity are excellent predictors of the presence of LC or HNC, with AUCs superior to 0.90 The analytical sensitivity and specificity of ARE and PON1 determinations were greater than 80% for both LC and HNC (Figure 1). Binary logistic regression analysis showed that PON1-related variables were associated with the presence or absence of disease, independent of differences in sex, age and clinical variables (Table 2 and Table 3).

**Figure 2:**
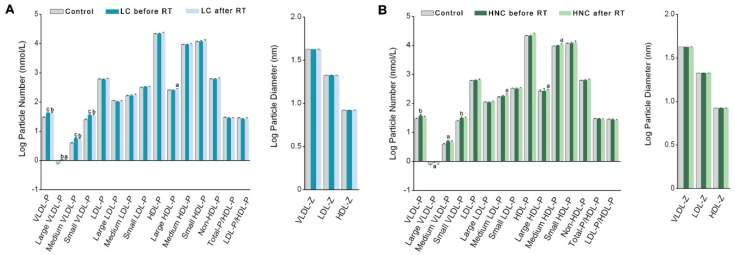
Number and diameter of the different lipoprotein particles in patients with lung cancer (LC) (**A**), head and neck cancer (HNC) (**B**) before and after radiotherapy (RT) and in the control group. Values are log transformed due to the high variability of particle sizes and numbers. Results are shown as means and standard errors. HDL: high-density lipoproteins; IDL: intermediate-density lipoproteins; LDL: low-density lipoproteins; VLDL: very-low-density lipoproteins. The suffix −P indicates number of particles, and the suffix −Z indicates the particle diameter. ^a^ *p* < 0.05, ^b^ *p* < 0.01, ^c^ *p* < 0.001, with respect to the control group by the Mann-Whitney *U*-test.

We found several changes in the pre-RT lipoprotein profile of the LC or HNC patients compared with the control group. Both types of patients showed a significant increase in the number of VLDL particles of all sizes, without changes in the number of particles of the other lipoproteins or in their diameters. Irradiation was not associated with any significant change in these parameters compared to pre-RT values (Figure 2).

LC patients had significantly higher VLDL- and IDL-cholesterol concentrations before RT, and higher triglycerides in all lipoprotein fractions. RT did not produce any significant changes in these variables (Table 4). HNC patients had significantly higher IDL-cholesterol concentrations before RT, and higher triglyceride levels in all lipoprotein fractions. In these patients, RT was associated with a significant increase in HDL-cholesterol concentrations (Table 5). This change was not due to an augmented cholesterol load per particle, since the ratios between cholesterol concentrations and the number of HDL particles was not significantly different before and after RT; therefore, it must be explained by an increase in the number of particles (Supplementary Table S1).

**Figure 3:**
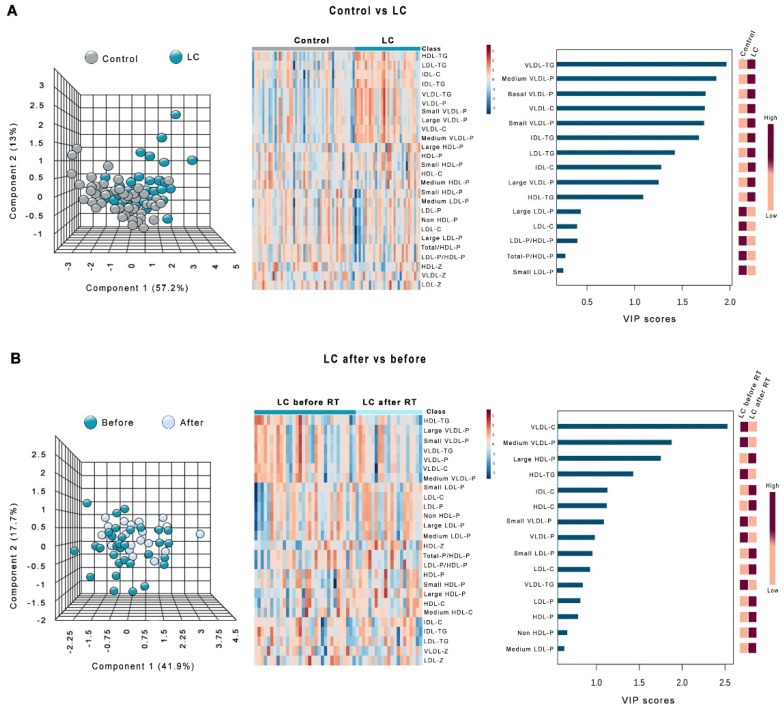
Principal component analysis (PCA) scores, heatmaps, and variable importance in projection (VIP) scores of the partial least squares discriminant analysis (PLSDA) in lung cancer (LC) patients vs. the control group (**A**) and in LC patients before and after radiotherapy (**B**). HDL: high-density lipoproteins; IDL: intermediate-density lipoproteins; LDL: low-density lipoproteins; VLDL: very-low-density lipoproteins. The suffix −P indicates number of particles, and the suffix −Z indicates the particle diameter.

**Figure 4:**
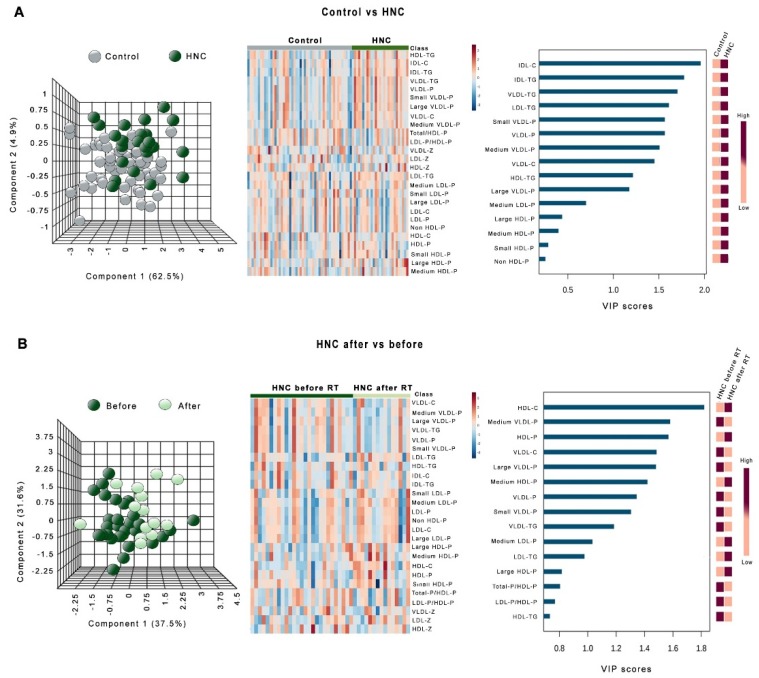
Principal component analysis (PCA) scores, heatmaps, and variable importance in projection (VIP) scores of the partial least squares discriminant analysis (PLSDA) in head and neck cancer (HNC) patients vs. the control group (**A**) and in HNC patients before and after radiotherapy (**B**). HDL: high-density lipoproteins; IDL: intermediate-density lipoproteins; LDL: low-density lipoproteins; VLDL: very-low-density lipoproteins. The suffix −P indicates number of particles, and the suffix −Z indicates the particle diameter.

Figure 3 shows the heatmaps, PCA, and PLDSA analyses of the changes in lipoprotein profile in LC patients. PCA did not show any global difference between these patients and the control group. To identify the most important lipoproteins related to LC, we evaluated VIP scores. This score measures modification of a variable associated with the disease state i.e., the higher the VIP score, the more relevant the classification. The VIP analysis identified VLDL-triglycerides as the most relevant components (Figure 3A). We also did not observe any significant global difference in the lipoprotein profile of LC patients before and after RT. In this case, VIP analysis identified VLDL-cholesterol as the most relevant component (Figure 3B). Very similar results were obtained in HNC patients, but in this disease, VIP score identified HDL-cholesterol as the component most strongly affected by RT (Figure 4).

Globally, differences in the pre-RT lipoprotein profile between LC and HNC patients and the control subjects were small and did not vary between groups. Multiple regression analysis showed that differences in age and smoking status may account for these alterations (Supplementary Table S2). We did not find any significant correlation (Spearman’s ρ test) between serum PON1 concentration or ARE activity and the lipoprotein-related variables (data not shown).

### 3.2. Relationships Between the Analytical Variables and the Clinical and Pathological Characteristics of the Patients

**Figure 5:**
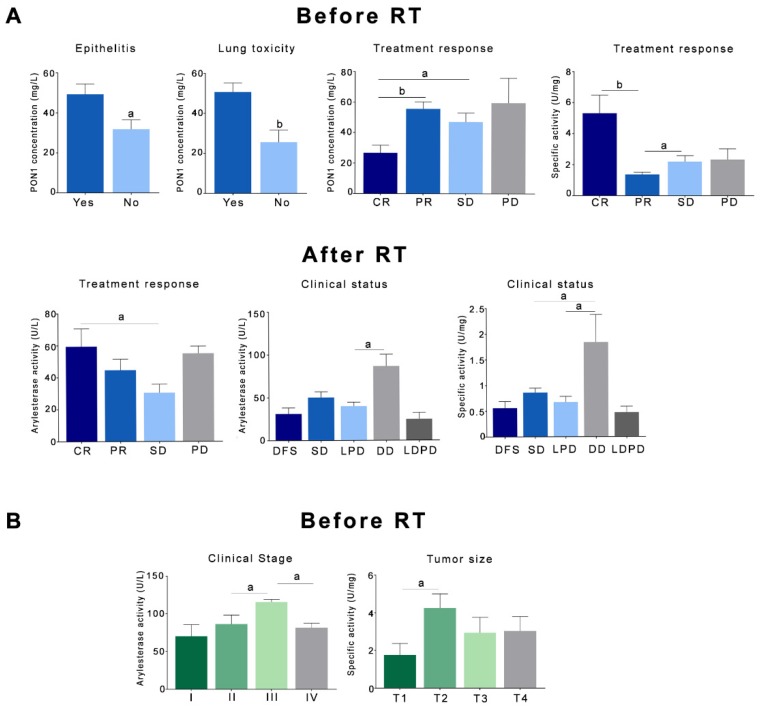
Relationships between paraoxonase-1-related variables and the toxicity to radiotherapy, clinical status, and response to treatment in patients with lung cancer (**A**) or head and neck cancer (**B**). Results are shown as means and standard errors. ^a^ *p* < 0.05, ^b^ *p* < 0.01, by the Mann-Whitney *U*-test. CR: complete response; DD: distant recurrence of the disease; DFS: disease-free survival; LDPD: loco-regional and distant progression of the disease; LPD: loco-regional progression of the disease; PD: progression of the disease. PON1: paraoxonase-1; PR: partial response; RT: radiotherapy; SD: stabilization of the disease.

Patients with LC who presented a CR to the treatment had significantly lower pre-RT PON1 concentrations and greater specific activities than the rest of the patients. Patients with stabilization of the disease had lower post-RT ARE activities. In relation to clinical status, patients with LC who presented a distant recurrence of their disease had significantly higher post-RT ARE activities and specific activities. Toxic reactions to RT, such as epithelitis and lung toxicity, were associated with lower PON1 concentrations in LC (Figure 5A). We did not find any significant association between toxicity and PON1-related variables in HNC, but higher ARE activities were found in patients with clinical stage III, as were higher specific activities in patients with intermediate-low size tumors (Figure 5B). Results are shown in detail in Supplementary Tables S3–S5.

## 4. Discussion

Several studies have reported decreased serum PON1 activity in LC and HNC [3,22,23,24,25,26]. This antioxidant enzyme is particularly abundant in epithelial tissue [27,28]. PON1 in lung tissue is localized in Clara cells, endothelial cells, and type I cells of the alveolar epithelium. Epithelial cells located in the respiratory portion of the lung or in the upper gastrointestinal tract can be exposed to tobacco smoke and reactive oxygen substances released by environmental toxicants [29]. Decreased serum PON1 activities in LC and HNC can be due to at least two factors which are not mutually exclusive: First, oxidative stress inhibits PON1 activity, since the PON1 active site for lipid peroxide hydrolysis requires a free sulfhydryl group at cysteine 284; PON1 degrades lipid peroxides by reacting covalently with this site which leading to enzyme inactivation [30]. Hence, the net result of increased oxidative stress is decreased PON1 activity. Second, the present study shows that the decrease in enzymatic activity is associated with a decrease in the serum concentration of the enzyme, suggesting an inhibition of PON1 hepatic synthesis. In this regard, LC and HNC differ from other types of cancer, such as breast cancer, in which we reported a decrease in serum PON1 activity [31], but without changes in concentration, and in other types of noncommunicable diseases associated with oxidative stress, in which an increase in the serum concentration of PON1 is often observed together with low enzyme activities [32].

The cause of these differences between diseases or types of cancer cannot be deduced from the present study, but it is feasible that different factors that induce cancer produce different effects in the synthesis of PON1. Indeed, it has already been described that some xenobiotics inhibit the hepatic synthesis of PON1, probably through the inhibition of peroxisome proliferator-activated receptor delta [33]. Therefore, the possibility exists that toxic xenobiotics present in tobacco smoke are causal factors of the decreased serum PON1 concentration.

Probably the most striking result of the present study is the high analytical sensitivity and specificity of the measurements of ARE activity and/or PON1 concentration in the discrimination between healthy subjects and patients with LC or HNC. Our results suggest that these determinations may be of great interest for the study of these two types of cancer. Actually, these results are logical, because cigarette smoke and its multiple ingredients have a strong carcinogenic activity, and while the mechanisms by which these carcinogenic agents act are complex and not fully understood, there is little doubt that oxidative damage and inflammation play a significant role [34,35]. Unfortunately, the epidemiological studies carried out so far lack adequate biomarkers for the study of oxidative stress in LC and HNC. Our study suggests that the determinations of ARE and/or PON1 can be useful markers for this purpose. Furthermore, as far as we know, this is the first study to investigate associations of the PON1-related variables with the clinical and pathological characteristics of the patients and their tumors and the response to treatment with RT. The finding of higher concentrations of PON1, together with unmodified ARE activities in patients who presented lung toxicity or epithelitis, suggest a compensatory attempt of the organism to increase the levels of this enzyme; this raises questions about the potential usefulness of the treatment with oral antioxidants for the reduction of these toxic reactions. Similarly, it is interesting to note that patients with LC who presented a CR had lower PON1 serum concentrations than those who presented a partial response or did not respond to RT; this could also be a compensatory increase in the synthesis of this enzyme in patients who do not respond correctly to treatment.

Obviously, our results should be validated and confirmed using wider series of patients and from different geographic locations. On the other hand, ARE and PON1 measurements are presently still in use for research purposes only, and have some drawbacks in the clinical routine: The determination of ARE is simple and economical, but the substrate is quite unstable and does not lack a certain toxicity. The determination of the concentration of PON1 can be carried out by ELISA and, although we employed an in-house method, several companies have commercialized reliable reagents. However, the determination of serum PON1 concentration with commercial kits is relatively expensive, and these methods have not been automated yet. We believe that if the analytical utility of serum ARE and PON1 is confirmed, simpler methods should be developed, through the development of biosensors or methods applicable to automatic analyzers, for their practical application in the daily clinic.

We observed several differences in the lipoprotein profile between healthy subjects and patients with LC or HNC. Our study is novel in that NMR methods allowed us to not only determine the concentrations of lipids in all the lipoprotein fractions, but also to observe the number of particles and their size. The most consistent alteration was a marked increase in triglyceride concentrations in all fractions, while LC patients had increased cholesterol levels in VLDL and IDL, and HNC patients, in IDL. This increase in VLDL-triglycerides was associated with an increase in the number of particles, but not in their size. These results show the presence of a pro-atherogenic lipoprotein profile in patients with LC or HNC. Several studies have investigated alterations in lipoprotein levels in these diseases. Decreased HDL-cholesterol concentrations have been reported in patients with oral cancer [36,37] or LC [11,38,39,40]. Our results differ from these studies. Also, studies differ on the existence of alterations in non-HDL lipoproteins in these types of cancer. We do not know the cause, but it is possible that differences in lifestyle, exposure to toxins, and the small number of patients investigated by most studies account in part for these discrepancies [41]. Moreover, our patients were older and had a higher proportion of men than the control group. Multiple regression analysis showed that differences in lipid parameters were not maintained when adjusted for age and sex. Notwithstanding the above, all researchers agree that the investigation of lipoprotein alterations in patients with LC or HNC is a subject of clinical importance, since these patients present a pro-atherogenic lipoprotein profile and, in addition, lipoproteins play an important role in tumor survival and progression, supplying triglycerides as fuel for energy metabolism and the cholesterol that enters in the composition of lipid rafts, which are key elements in the signaling pathways of tumor cells [11]. Changes in the apolipoprotein composition of HDL particles are likely to be found in cancer and/or irradiation, and will not necessarily be reflected in alterations in the cholesterol concentration. Sproull et al. [42,43] found that serum amyloid A (SAA) levels were increased in mice 24 h after irradiation, and suggested that SAA might be a useful biomarker for radiation exposure in a variety of total- and partial-body irradiation settings. In addition, a proteomic study from Wang el al. [44] reported that SAA may be a marker of pneumonitis in LC. Irradiation has also been reported to increase the levels of apolipoprotein E-enriched HDL in rats, while lipid peroxidation increased and vitamin E decreased [45]. Research on biomarkers regarding the efficacy of radiation therapy in cancer, as well as exposure levels and the associated toxic response, is an obvious clinical need [46]. Globally, studies suggest that the compositional changes of HDL particles is a field with great potential interest.

Information on the metabolic changes produced by RT in patients with LC or HNC is scarce. In the present study, we found an increase in the serum PON1 concentration with a decrease in ARE activity and a consequent decrease in specific activity. These results indicate a decrease in the antioxidant defenses that may suggest that the administration of oral antioxidants could be of clinical utility. We have only found one other article that investigated the changes in the activity of PON1 produced by RT in patients with HNC, which, similar to us, found an increase in oxidative stress and a decrease in the activities of several antioxidant enzymes, including PON1 [47].

## 5. Conclusions

We concluded that patients with LC or HNC presented a significant decrease in pre-RT serum ARE activity and PON1 concentration compared to healthy subjects. Conversely, changes observed in the lipoprotein profile were small and were not maintained when adjusted for age, sex, and other clinical and demographic variables. Our results suggest that determinations of the levels of PON1-related variables may constitute good biomarkers for the evaluation of these diseases. Studies with a larger number of patients are needed to confirm this hypothesis.

## Figures and Tables

**Table 1:** Clinical and demographic characteristics of the lung cancer and the head and neck cancer patients and the control group.

Clinical and Demographic Characteristics	Control Group *n* = 50	Lung Cancer *n* = 33	Head and Neck Cancer *n* = 28	*p*-Value *
Age, years	42 (35–47)	72 (65–79)	65 (56–73)	<0.001
Male sex, *n* (%)	25 (50.0)	26 (78.8)	25 (89.3)	<0.001
Alcohol habit (>20 g/day), *n* (%)	15 (30.0)	8 (24.2)	11 (39.3)	0.042
Smoking, *n* (%) Current smoker Former smoker	17 (34.0) 0 (0.0)	14 (42.4) 17 (51.5)	16 (57.1) 6 (21.4)	<0.001
Hypertension, *n* (%)	4 (8.0)	19 (57.6)	17 (60.7)	<0.001
Type 2 diabetes mellitus, *n* (%)	5 (10.0)	10 (30.3)	6 (21.4)	0.085
**Cancer stage, *n* (%)**				
Stage I	−	14 (42.4)	5 (17.9)	−
Stage II	−	4 (12.1)	5 (17.9)	−
Stage III	−	15 (45.5)	5 (17.9)	−
Stage IV	−	0 (0.0)	13 (46.3)	−
**Histology, *n* (%)**				
Squamous carcinoma	−	18 (54.5)	26 (92.9)	−
Adenocarcinoma	−	13 (39.4)	1 (3.6)	−
Others	−	2 (6.0)	1 (3.6)	−
**Chemotherapy treatment, *n* (%)**	−	18 (54.5)	17 (60.7)	−
**Secondary effects of radiotherapy, *n* (%)**				
Epithelitis	−	24 (72.7)	25 (89.3)	−
Lung toxicity	−	25 (75.8)	−	−
Xerostomia	−	−	22.0 (78.6)	−
Mucositis	−	−	20.0 (71.4)	−
Esophagitis	−	−	6.0 (21.5)	−

* Age is shown as medians and interquartile ranges (in parenthesis). Statistical analyses were performed by the χ-square test, except for age, that was done by the Mann-Whitney *U*-test.

**Table 2:** Binary logistic regression analyses of selected variables showing their significant and independent association or not with the presence or absence of cancer.

Model	B	Standard Error	*p*-Value	Exp (B)	95% IC for Exp (B)
Paraoxonase-1 concentration	−0.107	0.040	0.007	0.899	0.832–0.971
Age	0.205	0.097	0.035	1.227	1.015–1.484
Sex	−1.362	2.238	0.543	0.256	0.003–20.600
Smoking	4.107	1.963	0.036	60.794	1.296–2851.701
Arterial hypertension	0.321	2.753	0.907	1.378	0.006–303.664
Diabetes mellitus	−0.448	3.732	0.905	0.639	0.000–960.204
Constant	−5.486	6.553	0.402	0.004	−

**Table 3:** Binary logistic regression analyses of selected variables showing their significant and independent association or not with the presence or absence of cancer.

Model *	B	Standard Error	*p*-Value	Exp (B)	95% IC for Exp (B)
Arylesterase Activity	−0.037	0.013	0.005	0.964	0.939–0.989
Age	0.144	0.071	0.042	1.154	1.005–1.326
Sex	−2.033	2.060	0.324	0.131	0.002–7.428
Smoking	2.845	1.600	0.075	17.199	0.747–395.05
Arterial hypertension	1.605	2.841	0.572	4.977	0.019–1304.649
Diabetes mellitus	−1.385	2.437	0.570	0.250	0.002–29.732
Constant	−1.365	4.227	0.747	0.255	−

**Table 4:** Hematological variables and plasma cholesterol and triglycerides concentrations in lung cancer patients before and after radiotherapy.

Variable *	Control Group	Lung Cancer Before Radiotherapy	Lung Cancer After Radiotherapy
Hemoglobin (g/dL)	14.9 (13.9–15.2)	13.5 (11.0–13.9) ^c^	12.0 (11.3–13.1) ^c^
Platelets (×10^9^/L)	277.0 (230.5–301.5)	293.5 (241.0–357.0)	175.0 (138.5–202.0) ^c,d^
Total-cholesterol (mg/dL)	155.9 (140.2–172.8)	154.4 (135.6–177.0)	161.8 (139.9–178.9)
VLDL-cholesterol (mg/dL)	12.1 (8.6–16.7)	18.0 (13.6–21.7) ^b^	15.9 (10.3–20.3)
IDL-cholesterol (mg/dL)	7.3 (4.9–9.9)	8.9 (7.3–10.0) ^a^	10.0 (7.3–11.2) ^b^
LDL-cholesterol (mg/dL)	89.3 (80.3–100.5)	88.1 (68.9–99.4)	92.6 (69.3–106.6)
HDL-cholesterol (mg/dL)	43.4 (38.1–51.8)	43.6 (34.3–52.1)	47.1 (38.5–49.3)
Total-triglycerides (mg/dL)	69.3 (49.5–82.2)	97.3 (77.2–122.9) ^c^	94.0 (69.7–104.5) ^c^
VLDL-triglycerides (mg/dL)	38.1 (29.4–49.3)	64.0 (41.1–78.1) ^c^	60.3 (44.2–69.6) ^c^
IDL-triglycerides (mg/dL)	6.8 (4.9–8.3)	9.4 (7.7–10.2) ^c^	9.8 (7.4–11.0) ^c^
LDL-triglycerides (mg/dL)	8.8 (6.5–11.2)	11.6 (8.6–14.7) ^b^	11.0 (8.7–14.9) ^b^
HDL-triglycerides (mg/dL)	11.3 (8.7–15.3)	14.3 (11.8–17.9) ^b^	13.7 (11.9–14.9)

* Results are shown as medians and interquartile ranges (in parenthesis). HDL: high-density lipoproteins; IDL: intermediate-density lipoproteins; LDL: low-density lipoproteins; VLDL: very-low-density lipoproteins. ^a^ *p* < 0.05, ^b^ *p* < 0.01, ^c^ *p* < 0.001, with respect to the control group; ^d^ *p* < 0.001, with respect to before radiotherapy, by the Mann-Whitney *U*-test.

**Table 5:** Hematological variables and plasma cholesterol and triglycerides concentrations in head and neck cancer patients before and after radiotherapy.

Variable *	Control Group	Head and Neck Cancer Before Radiotherapy	Head and Neck Cancer After Radiotherapy
Hemoglobin (g/dL)	14.9 (13.9–15.2)	13.9 (11.8–14.8)	11.2 (10.3–12.4) ^c,d^
Platelets (×10^9^/L)	277.0 (230.5–301.5)	208.0 (154.5–327.0)	195.0 (161.0–277.0) ^a^
Total-cholesterol (mg/dL)	155.9 (140.2–172.8)	157.8 (140.3–176.0)	164.9 (151.4–206.5)
VLDL-cholesterol (mg/dL)	12.1 (8.6–16.7)	14.9 (10.8–19.7)	12.2 (8.2–17.5)
IDL-cholesterol (mg/dL)	7.3 (4.9–9.9)	8.5 (6.7–11.8) ^a^	9.7 (6.6–10.3)
LDL-cholesterol (mg/dL)	89.3 (80.3–100.5)	93.0 (76.8–100.5)	99.8 (78.0–110.2)
HDL-cholesterol (mg/dL)	43.4 (38.1–51.8)	42.6 (38.9–46.6)	52.1 (44.8–57.2) ^a^
Total-triglycerides (mg/dL)	69.3 (49.5–82.2)	89.5 (67.8–96.8) ^b^	78.1 (64.8–88.4) ^a^
VLDL-triglycerides (mg/dL)	38.1 (29.4–49.3)	54.5 (42.5–62.7) ^b^	46.3 (33.9–52.0)
IDL-triglycerides (mg/dL)	6.8 (4.9–8.3)	8.4 (7.5–10.7) ^b^	8.9 (7.5–9.6) ^b^
LDL-triglycerides (mg/dL)	8.8 (6.5–11.2)	11.2 (9.3–11.7) ^a^	12.7 (10.5–14.2)
HDL-triglycerides (mg/dL)	11.3 (8.7–15.3)	13.1 (11.1–17.6) ^a^	12.0 (10.6–17.0)

* Results are shown as medians and interquartile ranges (in parenthesis). HDL: high-density lipoproteins; IDL: intermediate-density lipoproteins; LDL: low-density lipoproteins; VLDL: very-low-density lipoproteins. ^a^ *p* < 0.05, ^b^ *p* < 0.01, ^c^ *p* < 0.001, with respect to the control group; ^d^ *p* < 0.05 with respect to before radiotherapy, by the Mann-Whitney *U*-test.

## Supplementary Materials

The following are available online at https://www.mdpi.com/2076-3921/8/7/213/s1, Table S1: Ratios between the concentration of cholesterol in high-density lipoproteins and the number of particles before and after radiotherapy. Table S2: Multiple regression analyses of selected variables showing their significant and independent association or not with the presence or absence of cancer. Table S3: Relationships between paraoxonase-1-related variables before radiotherapy and toxic reactions to radiation, clinical status, and response to treatment in patients with lung cancer. Table S4: Relationships between paraoxonase-1-related variables after radiotherapy and toxic reactions to radiation, clinical status, and response to treatment in patients with lung cancer. Table S5: Relationships between paraoxonase-1-related variables and the toxicity to radiotherapy, clinical status, and response to treatment in patients with head and neck cancer.
