# Supplementary material for: Serum Paraoxonase-1-Related Variables and Lipoprotein Profile in Patients with Lung or Head and Neck Cancer: Effect of Radiotherapy
Source: Antioxidants (Basel). 2019 Jul 10;8(7):213. doi: 10.3390/antiox8070213 (PMC6680864; doi:10.3390/antiox8070213)
Supplement: Supplementary file 1 [file antioxidants-08-00213-s001.pdf]

Supplementary Table 1. Ratios between the concentration of cholesterol in high-density lipoproteins and the number of particles before and after radiotherapy

Variable	**Before radiotherapy**	**After radiotherapy**	**p-value**
HDL-C/HDL-P large	163.9 (141.2-181.1)	175.5 (145.6-198.9)	0.168
HDL-C/HDL-P medium	4.6 (4.0-4.8)	4.8 (4.3-5.4)	0.312
HDL-C/HDL-P small	3.3 (3.1-4.1)	3.4 (3.2-4.1)	0.470

HDL-C: Cholesterol concentration in high-density lipoproteins. HDL-P: Number of high-density lipoprotein particles (large, medium and small). Statistical comparisons were done with the Mann-Whitney *U*-test.

Supplementary Table 2. Multiple regression analyses of selected variables showing their significant and independent association or not with the presence or absence of cancer

Model*	β coefficient	Standard error	t	p-value
Constant	2.696	0.185	14.559	< 0.001
Age	-0.018	0.002	-7.854	< 0.001
Sex	0.095	0.076	1.248	0.215
Smoking	-0.091	0.043	-2.098	0.038
Alcohol intake	-0.010	0.035	-0.281	0.779
Hypertension	-0.030	0.060	-0.498	0.620
Type 2 diabetes mellitus	0.031	0.058	0.527	0.600
VLDL-cholesterol	0.015	0.010	1.476	0.143
VLDL-triglycerides	-0.005	0.003	-1.762	0.081
IDL-triglycerides	-0.026	0.031	-0.856	0.394
LDL-triglycerides	-0.003	0.014	-2.225	0.822
HDL-triglycerides	0.003	0.016	0.166	0.869

*Model summary: $r^2 = 0.674$; $p < 0.001$. HDL: high-density lipoprotein; IDL: intermediate-density lipoprotein; LDL: low-density lipoprotein; PON1: paraoxonase-1; VLDL: very-low-density lipoprotein.

Supplementary Table 3. Relationships between paraoxonase-1-related variables before radiotherapy and toxic reactions to radiation, clinical status, and response to treatment in patients with lung cancer

	**Before radiotherapy**		
**Variable**	**Paraoxonase-1 (mg/L)**	**Arylesterase (U/L)**	**Specific activity (U/mg)**
**Epithelitis**			
Yes (n=24)	50.1 (28.8-59.6)	93.8 (69.6-103.4)	1.5 (1.2-3.8)
No (n=9)	32.8 (20.1-44.0)[a]	97.3 (59.2-101.6)	2.4 (1.6-6.6)
**Lung toxicity**			
Yes (n=25)	49.7 (34.4-59.1)	93.4 (66.8-100.4)	1.6 (1.2-2.7)
No (n=8)	20.9 (10.1-44.2)[b]	101.6 (64.5-102.5)	6.3 (1.6-10.1)
**Treatment response**			
Complete response (n=11)	25.4 (10.5-43.2)	101.0 (75.1-102.4)	4.0 (1.6-9.8)[f]
Partial response (n=10)	54.8 (47.3-61.4)[d]	72.9 (59.4-94.4)	1.2 (0.8-1.7)
Stabilization of disease (n=7)	51.2 (34.5-60.2)[c]	96.5 (75.1-103.8)	1.8 (1.5-3.3)[e]
Progression of disease (n=5)	41.8 (29.3-98.6)	99.4 (71.4-121.8)	2.4 (0.9-3.7)
**Clinical status**			
Disease free survival (n=4)	27.8 (14.0-50.1)	100.1 (64.1-103.4)	2.9 (1.7-8.3)
Stabilization of disease (n=8)	50.8 (40.5-56.2)	66.8 (49.3-91.0)	1.4 (0.9-1.8)
Locoregional progression of disease (n=8)	48.4 (37.9-59.7)	79.9 (75.1-103.6)	1.5 (1.3-2.1)
Distant recurrence of disease (n=5)	32.8 (17.7-42.5)	99.4 (73.2-101.9)	3.0 (1.7-7.1)
Locoregional and distant progression of disease (n=6)	60.0 (23.7-76.7)	95.2 (84.0-118.8)	1.4 (0.8-4.7)
Unknown (n=2)	33.8 (29.7)	113.2 (102.5)	3.4 (3.3)

The table only shows the clinical characteristics significantly associated with changes in PON1-related variables in at least one subgroup.
Results are shown as medians and interquartile ranges (in parenthesis). [a] $p < 0.05$, [b] $p < 0.01$, with respect to Yes; [c] $p < 0.05$, [d] $p < 0.01$, with respect to complete response; [e] $p < 0.05$, [f] $p < 0.01$, with respect to partial response. Statistical comparisons were done with the Mann-Whitney *U*-test.

Supplementary Table 4. Relationships between paraoxonase-1-related variables after radiotherapy and toxic reactions to radiation, clinical status, and response to treatment in patients with lung cancer

	After radiotherapy		
**Variable**	**Paraoxonase-1 (mg/L)**	**Arylesterase (U/L)**	**Specific activity (U/mg)**
**Epithelitis**			
Yes (n=17)	55.4 (47.1-71.4)	42.8 (30.1-61.9)	0.8 (0.4-1.0)
No (n=5)	54.7 (48.7-62.0)	43.1 (33.3-57.3)	0.8 (0.5-1.1)
**Lung toxicity**			
Yes (n=16)	55.8 (46.1-69.7)	42.4 (29.3-48.7)	0.7 (0.4-1.0)
No (n=6)	54.6 (48.7-61.3)	52.8 (38.2-80.7)	0.9 (0.7-1.5)
**Treatment response**			
Complete response (n=7)	54.5 (45.1-66.3)	50.5 (43.1-97.8)	0.9 (0.6-1.3)
Partial response (n=8)	68.8 (54.9-82.3)	42.4 (29.5-65.7)	0.7 (0.4-1.0)
Stabilization of disease (n=5)	52.9 (46.2-58.8)	31.7 (18.4-42.1)[c]	0.7 (0.3-0.8)
Progression of disease (n=2)	49.8 (44.3)	55.1 (50.5)	1.1 (0.9)
**Clinical status**			
Disease free survival (n=3)	61.5 (43.2)	31.7 (19.2)	0.6 (0.3)
Stabilization of disease (n=7)	50.8 (40.5-56.2)	43.1 (42.8-73.3)	0.9 (0.8-1.0)
Locoregional progression of disease (n=7)	57.8 (52.9-85.7)	42.0 (28.5-50.5)	0.8 (0.4-0.9)
Distant recurrence of disease (n=3)	44.3 (35.4)	97.8 (59.6)[a]	1.3 (1.3)[a,b]
Locoregional and distant progression of disease (n=2)	52.3 (49.2)	25.1 (17.5)	0.5 (0.4)

The table only shows the clinical characteristics significantly associated with changes in PON1-related variables in at least one subgroup. Results are shown as medians and interquartile ranges (in parenthesis). [a] $p < 0.05$, with respect to locoregional progression of disease; [b] $p < 0.05$, with respect to stabilization of disease. Statistical comparisons were done with the Mann-Whitney *U*-test.

Supplementary Table 5. Relationships between paraoxonase-1-related variables and the toxicity to radiotherapy, clinical status, and response to treatment in patients with head and neck cancer

	Before radiotherapy	
**Variable**	**Arylesterase (U/L)**	**Specific activity (U/mg)**
**Clinical stage**		
I (n=5)	51.8 (32.9-95.0)	1.0 (0.6-2.4)
II (n=5)	99.2 (62.2-104.4)[a]	3.4 (2.8-6.0)
III (n=5)	118.2 (111.4-120.2)	2.7 (1.1-4.6)
IV (n=13)	79.6 (64.9-90.0)[a]	2.1 (1.4-5.3)
**Tumor size**		
T1 (n=4)	67.2 (42.1-101.8)	1.5 (0.8-3.0)
T2 (n=9)	101.5 (80.0-112.0)	3.1 (2.6-6.5)[b]
T3 (n=7)	82.2 (63.9-119.7)	1.8 (1.1-3.9)
T4 (n=6)	84.3 (76.2-96.1)	2.2 (1.6-5.0)
Unknown (n=2)	41.9 (26.9)	0.9 (0.6)

The table only shows the clinical characteristics significantly associated with changes in PON1-related variables in at least one subgroup. Results are shown as medians and interquartile ranges (in parenthesis). [a] $p < 0.05$, with respect to clinical stage III; [b] $p < 0.05$, with respect to T1. Statistical comparisons were done with the Mann-Whitney *U*-test.
